# Rituximab for refractory ocular symptoms in myasthenia gravis with thyroid eye disease: a case report with quantitative MRI assessment

**DOI:** 10.3389/fimmu.2025.1717260

**Published:** 2025-11-27

**Authors:** Dingxian He, Xiangwen Li, Baitong Wang, Yaqi Wang, Chong Yan, Mengxiao Liu, Tom Hibert, Sushan Luo, Chongbo Zhao, Jianying Xi

**Affiliations:** 1 Huashan Rare Disease Centre and Department of Neurology, Huashan Hospital, Shanghai Medical College, National Centre for Neurological Disorders, Fudan University, Shanghai, China; 2 Department of Radiology, Huashan Hospital Fudan University, Shanghai, China; 3 Department of Encephalopathy, The Affiliated Hospital to Changchun University of Chinese Medicine, Jilin, Changchun, China; 4 Magnetic Resonance (MR) Collaborations, Siemens Healthineers Ltd., Shanghai, China; 5 Swiss Innovation Hub, Siemens Healthineers International Aktiengesellschaft (AG), Lausanne, Switzerland

**Keywords:** myasthenia gravis, rituximab, thyroid eye disease, ocular symptoms, magnetic resonance imaging

## Abstract

**Aims:**

To report a case with concurrent myasthenia gravis (MG) and thyroid eye disease (TED) showing response to rituximab (RTX) through both clinical evaluation and quantitative MRI assessment.

**Methods:**

A 31-year-old female patient with refractory ocular symptoms from concurrent MG and TED received RTX after failing multiple conventional treatments. Treatment response was evaluated through clinical scores and quantitative T2 mapping of extraocular muscles (EOMs) before and after RTX administration.

**Results:**

Following RTX treatment, the patient achieved corticosteroid-free remission by six months post-RTX, with Myasthenia Gravis Activities of Daily Living score decreasing from 5 to 0, complete resolution of ptosis, and improvement in ocular motility and exophthalmos. Quantitative T2 mapping demonstrated significant reductions in T2 relaxation times (10.6%-20.9%) across all EOM regions of interest, with decreased standard deviations indicating restored tissue homogeneity.

**Conclusions:**

This case suggests potential therapeutic benefit of RTX for refractory ocular symptoms in concurrent MG and TED. Quantitative T2 mapping revealed pathologically elevated values that normalized post-treatment, providing preliminary objective evidence of treatment response. As a single case, the relative contributions of MG versus TED pathology cannot be definitively separated. Integrating clinical assessment with quantitative T2 mapping may offer an objective approach for monitoring treatment response in such complex dual autoimmune disorders.

## 1 Introduction

Myasthenia gravis (MG) is a prototypical B-cell-mediated autoimmune disease characterised by fluctuating skeletal muscle fatigue and weakness (1). The global incidence ranges from 0.3 to 2.8 per 100, 000 person-years, with prominent extraocular muscle (EOM) involvement (2). Nearly 50% of patients initially present with only ocular symptoms and 90% develop EOM dysfunction during disease progression (3). Despite significant advances in immunotherapy improving overall MG prognosis, ocular symptoms remain challenging to treat, with approximately 10% of patients experiencing persistent ptosis, restricted ocular mobility, or diplopia, substantially impairing health-related quality of life (4–6).

The coexistence of MG with thyroid eye disease (TED) presents particularly complex management challenges. This overlap is clinically significant, as the prevalence of thyroid disease in generalized MG ranges from 7-18%, with approximately 40% of ocular MG patients having antithyroid antibodies (7). Both conditions share molecular pathways involving antibody cross-reactivity, defective immune tolerance, and genetic susceptibilities that promote inflammation and fibrosis of EOMs (7). Current therapeutic approaches have substantial limitations in this dual pathology. Acetylcholinesterase inhibitors target only neuromuscular junction dysfunction, corticosteroids carry significant long-term side effects, and nonsteroidal immunosuppressants (NIST) require a long time to take effect and some patients may show no response (8–11).

Rituximab (RTX), a chimeric anti-CD20 monoclonal antibody, offers a promising therapeutic approach by targeting the shared B-cell-mediated pathogenesis in both conditions. Through selective depletion of immature, naive, and memory B cells while sparing plasma cells, RTX reduces autoantibody production and antigen presentation (12). Clinical evidence supports its efficacy in both refractory MG and TED, with RTX being recommended as a treatment option for refractory patients in the clinical guidelines for both conditions (2, 13). Therefore, RTX represents a promising dual therapeutic approach for patients with concurrent MG and TED.

In this study, we present a case utilizing quantitative magnetic resonance imaging (MRI) mapping combined with clinical assessment to evaluate RTX therapeutic response in concurrent refractory myasthenic ocular symptoms and TED. This case provides novel insights into objective treatment monitoring for this complex dual pathology and contributes evidence-based guidance for managing such challenging presentations.

## 2 Case description

### 2.1 Clinical history

A 31-year-old woman presented in late 2018 with alternating bilateral ptosis, diplopia, and impaired ocular motility (**Table 1**). MG was confirmed through repetitive nerve stimulation testing and positive for acetylcholine receptor antibody (AChR-ab). Initial treatment with oral prednisone (Pred, maximum dose 30 mg/d) and pyridostigmine (180mg/d) achieved minimal symptom expression (MSE). After initial remission, the patient experienced annual relapses triggered by stress, infections, or steroid tapering, each requiring intravenous methylprednisolone (IVMP) for symptom control. In 2020, the patient was diagnosed with concurrent hyperthyroidism (TSH↓0.03 mIU/L, FT3 28.55 pmol/L, FT4↑29.94 pmol/L, TRAb↑7.8IU/L, TPOAb↑185U/mL) and TED (CAS 4/7, NOSPECS class IV), presenting as bilateral exophthalmos that exacerbated existing ocular symptoms. Hyperthyroidism was managed with methimazole, achieving euthyroid status within 6 months. Multiple NISTs (tacrolimus and mycophenolate mofetil) were trialed during 2020–2021 with limited efficacy due to adverse effects or insufficient response.

**Table 1:** Clinical characteristics at RTX initiation.

Features	Patient
Sex	F
Age (years)	31
Age of MG onset (years)	26
Age of TED onset (years)	29
MGFA classification at maximal worsening	IIa
Duration of residual ocular symptoms (months)	17
Past immunotherapy	Pred, IVMP, TCR, MMF, IVIg, EFG
Current immunotherapy	Pred
EFG cycles completed	2
Time from last EFG to RTX (months)	6
Thymus condition	Normal
Thymectomy	(-)
AChR-ab	(+)
MuSK-ab	(-)
Thyroid function	
FT3 (pmol/L) [Normal: 3.1-6.8]	15.9
FT4 (pmol/L) [Normal: 12-22]	4.47
TSH (mIU/L) [Normal: 0.27-4.2]	0.73
TRAb (IU/L) [Normal: <1.75]	1.16
TPOAb (U/mL) [Normal: <34]	161 ↑
Present residual ocular symptoms	Ptosis, Diplopia, Limitation in eye motility
Concomitant disease	Hyperthyroidism, Hypertension, Diabetes, Postsplenectomy

MG, myasthenia gravis; TED, Thyroid eye disease; MGFA, Myasthenia Gravis Foundation of America; Pred, Prednisone; IVMP, Intravenous methylprednisolone; TCR, tacrolimus; MMF, mycophenolate mofetil; IVIg, Intravenous immunoglobulin; EFG, Efgartigimod; RTX, Rituximab; FT3, Free triiodothyronine; FT4, Free thyroxine; TSH, Thyroid stimulating hormone; TRAb, TSH Receptor Antibody; TPOAb, Thyroid peroxidase antibody; AChR-ab, acetylcholine receptor antibody.

An acute pneumonia in December 2022 caused disease exacerbation with generalized weakness (MGFA IIa). Subsequently, an upper respiratory infection in November 2023 triggered severe symptom recurrence with complete bilateral ptosis and multidirectional diplopia. Intravenous immunoglobulin (IVIg) was discontinued due to poor response and fever. As previously reported by our group (14), efgartigimod (EFG) treatment initiated in early 2024 provided only transient improvement, with symptom relapse occurring three weeks after completion.

### 2.2 Treatment course

**Figure 1:**
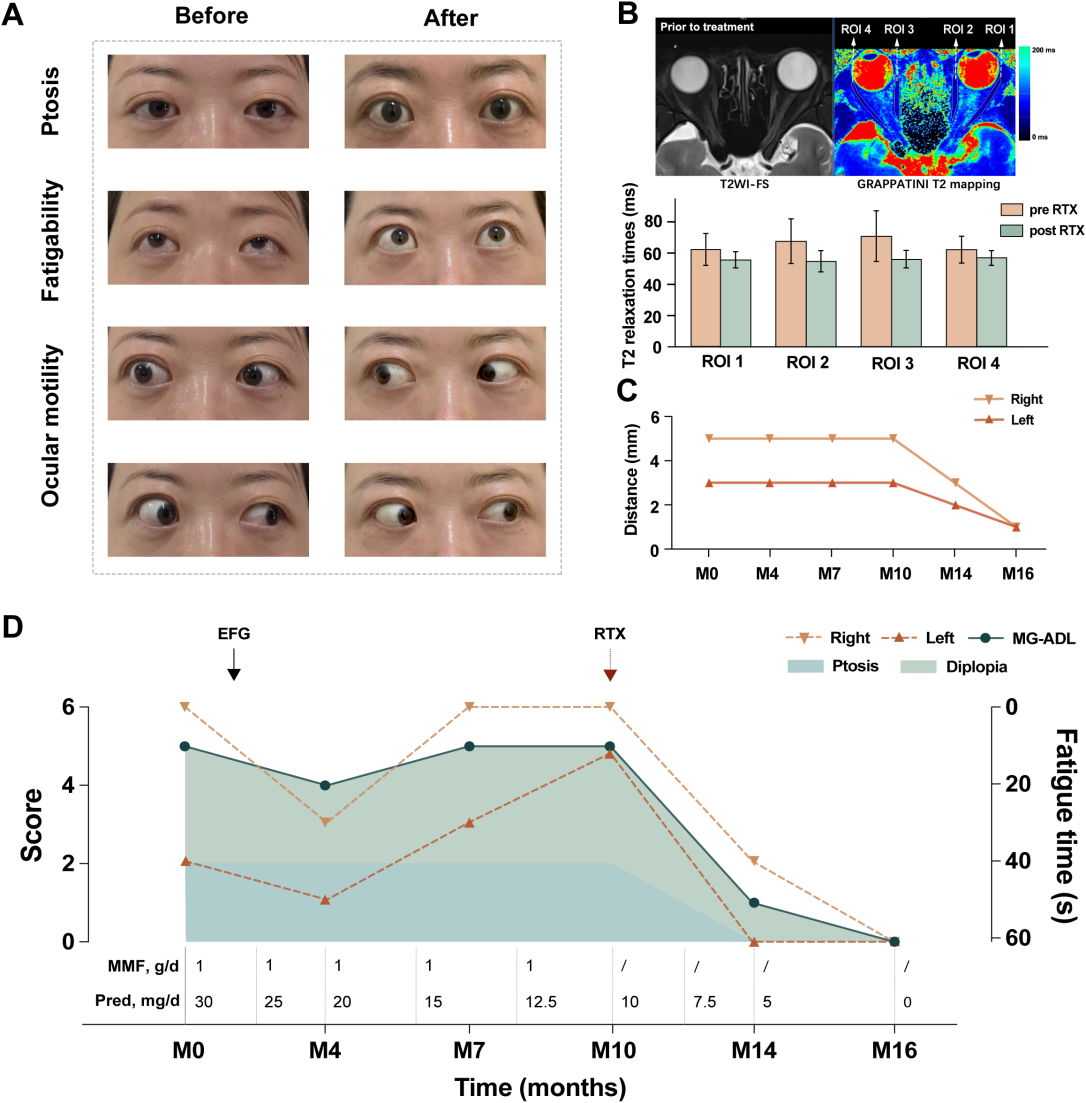
Rituximab treatment response in concurrent MG and TED. **(A)** Representative photographs demonstrating clinical improvements in ptosis, eyelid fatigue, and ocular motility before and after RTX treatment. **(B)** MRI evaluation using T2WI-FS sequences and GRAPPATINI T2 mapping demonstrating four ROIs prior to treatment. Bar graph displays quantitative T2 relaxation times (ms) for each ROI before (pre-RTX) and after (post-RTX) treatment. **(C)** Quantitative assessment of horizontal ocular motility (measured in millimeters) for right and left eyes over 16 months of follow-up. **(D)** Comprehensive treatment timeline illustrating MG-ADL scores, fatigue time, ptosis severity, diplopia, and concurrent medications (MMF and Pred dosages). Arrows indicate administration timepoints for EFG and RTX. MG, myasthenia gravis; TED, thyroid eye disease; T2WI-FS, T2-weighted imaging with fat suppression; MG-ADL, Myasthenia Gravis Activities of Daily Living; ROI, Region of interest; Pred, Prednisone; MMF, mycophenolate mofetil; RTX, Rituximab; EFG, Efgartigimod; EOM, Extraocular muscle.

We defined the time point of symptom exacerbation after previous treatment as M0, with the treatment flowchart illustrated in **Figure 1D**. Given the refractory ocular symptoms including persistent bilateral ptosis, multidirectional diplopia, severely restricted ocular motility and exophthalmos after oral medication adjustment, RTX was initiated after one cycle of EFG treatment (M10). The patient received RTX using a modified protocol: 100mg on day 1 followed by 500mg on day 2, with concurrent MMF discontinuation. Symptoms began improving at two months post-RTX, achieving MSE and Myasthenia Gravis Activities of Daily Living (MG-ADL) score of 1 by four months. Oral Pred was successfully discontinued at six months post-RTX, while thyroid function remained normal throughout the treatment course.

### 2.3 Clinical assessment and response

#### 2.3.1 Photograph and clinical score

At M0, the patient presented with severe ocular symptoms, evidenced by a MG-ADL score of 5 points, comprising 2 points for ptosis and 3 points for diplopia respectively, with bilateral horizontal ocular motility restricted to 5mm (right) and 3mm (left) (**Figures 1C, D**). Following one cycle of EFG treatment, mild improvement in eyelid fatigability was observed at M4, with improved fatigability time (**Figures 1A, D**). Progressive improvement became evident following RTX administration from M10 onwards (**Figure 1A**). By M16, clinical assessments demonstrated remarkable improvement across all parameters: the MG-ADL score decreased to 0, fatigability time of both eyelids extended beyond 60 seconds, and bilateral horizontal ocular motility limitation improved to 1mm (**Figures 1C, D**). Meanwhile, photographs demonstrated significant improvement in ptosis, ocular motility, and exophthalmos (**Figure 1A**). These results indicate substantial clinical improvement across all domains, with the patient achieving corticosteroid-free remission in MG and TED (CAS 0/10, NOSPECS class I).

#### 2.3.2 Quantitative MRI assessment

MRI examination was performed on a 3.0T MRI scanner (MAGNETOM Prisma; Siemens Healthcare, Erlangen, Germany). The MRI examination sequences for the EOMs include conventional T1-weighted imaging (T1WI), T2-weighted imaging (T2WI), and GRAPPATINI T2 mapping sequences. The detailed imaging parameters are summarized in **Table 2**. GRAPPATINI was performed with an undersampling factor of 5 combined with 2-fold GRAPPA undersampling, resulting in a 10-fold undersampling of the k-space data. Strong fat saturation is used for fat suppression. During the data preprocessing phase, the undersampled k-space missing data is first reconstructed using the GRAPPA algorithm, followed by signal normalization to eliminate equipment gain variations across different TEs. Gaussian filtering is applied to remove background orbital noise (e.g., signals from air and skull regions), and B_1_ field shimming correction is performed to optimize the local magnetic field homogeneity in the orbit, reducing signal deviations caused by magnetic field inhomogeneity near the EOM attachments.

**Table 2:** Overview of magnetic resonance sequence parameters.

	T1WI TSE	T2WI TSE	T2WI TSE	GRAPPATINI
Plane	Axial	Axial	Coronal	Axial
Time to repetition, ms	604	3120	3120	3500
Time to echo, ms	9.3	83	83	13.5, 27.0, 40.5, 54.0, 67.5, 81.0, 94.5, 108.0, 121.5, 135
No. slices	20	20	15	20
Slice thickness, mm	3	3	3	3
Field of view, mm^2^	200×200	200×200	180×180	180×180
Acquisition matrix	224×320	224×320	224×320	197×212
Voxel size, mm^3^	0.6×0.6×3.0	0.6×0.6×3.0	0.6×0.6×3.0	0.7×0.7×4.0
Phase encoding direction	R >> L	R >> L	R >> L	A >> P
Averages	1	1	1	2
Flip angle, degrees	160	160	160	180
Bandwidth, Hz/Px	391	391	381	200
Acquisition time, min:s	01:12	02:14	02:14	04:05

TSE, turbo spin echo; H, head; F, feet; A, anterior; P, posterior.

T2 value quantification employs nonlinear iterative fitting constrained by the MARTINI algorithm: Based on the mono-exponential decay model ( $S=M_{0}e^{-TE/T2}$), initial values for the T2 and equilibrium magnetization ( $M_{0}$) of the EOM tissue are assumed and substituted into the model to generate simulated signals. The Levenberg-Marquardt algorithm iteratively compares the residuals between the simulated and actually acquired signals. Concurrently, the residuals are constrained to conform to the physiological relaxation characteristics of EOM (reference T2 range: 40–60 msec) to prevent outliers, ultimately producing the quantitative T2 map. The region of interest (ROI) was manually drawn on the maximum central slice of the EOMs in T2W images. The ROI should be as large as possible to cover the entire muscle belly, but 1 mm away from the edge of the muscle belly to avoid the partial volume effect. Subsequently, the ROIs were copied to the T2 maps for T2 value measurement.

Serial evaluations were performed before and after RTX (**Figure 1B**). Prior to RTX treatment, T2-weighted fat-suppressed sequences revealed hyperintense signals in the EOM regions, corresponding to pathologically elevated T2 relaxation times (mean ± SD) on GRAPPATINI T2 mapping: region of interest 1 (ROI 1) 62.3 ± 10.2 ms, ROI 2 67.6 ± 14.3 ms, ROI 3 70.9 ± 16.2 ms, and ROI 4 62.2 ± 8.6 ms. Following RTX treatment, quantitative T2 mapping demonstrated significantly reduced T2 relaxation times across all ROIs (mean ± SD): ROI 1 55.7 ± 5.2 ms, ROI 2 54.8 ± 6.8 ms, ROI 3 56.1 ± 5.6 ms, and ROI 4 56.9 ± 4.7 ms. Notably, the standard deviations were substantially reduced in all regions, reflecting improved tissue homogeneity. The percentage reductions in T2 values were: ROI 1: 10.6%, ROI 2: 18.9%, ROI 3: 20.9%, and ROI 4: 8.5%. These quantitative improvements in T2 relaxation times, coupled with enhanced signal uniformity, indicate effective resolution of muscle inflammation and edema following RTX therapy. These quantitative imaging findings correlated with the patient’s clinical improvement, providing evidence of RTX therapeutic efficacy.

## 3 Discussion

Treating and assessing residual ocular symptoms in MG patients with concurrent TED remains challenging. We here report a case integrating clinical evaluation with quantitative MRI mapping to assess RTX efficacy in this complex dual pathology. RTX achieved remarkable therapeutic success, resulting in MSE and complete steroid withdrawal.

RTX demonstrated superior therapeutic advantages compared to conventional treatments. The patient experienced inadequate symptom control and recurrent exacerbations following treatment with multiple NISTs and the FcRn antagonist, necessitating repeated IVMP for symptom management. Consistent with our previous findings, while EFG provided rapid symptomatic improvement, its therapeutic effect was transient and failed to achieve complete remission of diplopia and ocular motility disorders, requiring continued oral NISTs therapy for maintenance (14). In contrast, RTX demonstrated comprehensive and sustained therapeutic benefits, significantly improving diplopia and ocular motility function while enabling complete steroid withdrawal.

The improvement in ocular symptoms may be attributed to the immunomodulatory effects of RTX through multiple pathways. In patients with MG complicated by TED, both diseases involve dysregulated autoimmune responses targeting different antigenic systems. By selectively depleting CD20+ B cells, RTX reduces production of pathogenic autoantibodies (AChR-abs and thyroid-stimulating immunoglobulins) while potentially disrupting cross-reactive immune responses between orbital and thyroid antigens (7, 15). As secondary effects, the reduction in inflammatory cytokines including IL-21 may decrease autoreactive follicular helper T cell activity and promote regulatory T cell expansion, facilitating immune system rebalancing (16). Through this comprehensive immune modulation, RTX may control autoimmune inflammatory processes affecting EOMs and improve muscle function coordination. However, in this dual autoimmune disorder case, the relative contributions of MG versus TED pathology to the observed EOM changes and therapeutic response cannot be definitively separated. The clinical improvements following RTX treatment likely reflect combined effects on both disease processes, though the predominant mechanism remains uncertain.

T2 mapping is a well-established quantitative imaging technique sensitive to collagen fiber organization and tissue water content, with validated feasibility for peripheral nerve and muscle assessment (17, 18). Traditional assessment of EOMs relies primarily on clinical manifestations, which are limited by high subjectivity and difficulty in objective quantification. In this case, the patient presented with typical clinical features of TED, including exophthalmos and eyelid retraction. Notably, visual assessment in this case did not reveal obvious EOM morphological enlargement, which does not contradict the clinical diagnosis, as while EOM hypertrophy is a common imaging finding in TED, it is not a necessary criterion for diagnosis. Normal EOM dimensions exhibit substantial inter-individual variability, and establishing abnormal thickening requires validated normative data, which remains limited. Despite the absence of obvious morphological changes, quantitative T2 mapping detected pathologically elevated T2 values (ROI 1: 62.3 ± 10.2 ms, ROI 2: 67.6 ± 14.3 ms, ROI 3: 70.9 ± 16.2 ms, ROI 4: 62.2 ± 8.6 ms) compared to normal EOM T2 values (approximately 40–45 ms), providing objective evidence of tissue abnormality. This suggests that T2 mapping may provide objective data independent of visual interpretation of morphological changes, potentially offering enhanced sensitivity in detecting early or subtle EOM pathology. Therapeutic response was clearly demonstrated through significant T2 relaxation time changes that objectively reflected resolution of inflammatory edema. In this case, the SD of ROIs decreased substantially from 8.6-16.2 ms to 4.7-6.8 ms after RTX, indicating restored tissue homogeneity and correlating with improved muscle function. This concordant improvement in imaging parameters and clinical symptoms in this individual case suggests the potential value of T2 mapping as a biomarker for treatment monitoring in complex cases where multiple pathological processes may coexist. However, caution is warranted in therapeutic interpretation, as the observed imaging changes likely reflect combined pathological effects of both MG and TED.

This study has several limitations: (1) As a single case report, generalizability requires validation through larger and further in-depth studies; (2) The short follow-up period necessitates longer-term observation to establish sustained efficacy and safety. (3) The lack of age- and sex-matched control data for EOM morphometric comparison limits our ability to definitively establish the presence and extent of typical TED-associated EOM hypertrophy; (4) In this dual autoimmune disorder, the relative therapeutic contributions of RTX to MG versus TED pathology cannot be definitively distinguished, as both conditions may contribute to EOM dysfunction and respond to B-cell depletion therapy. Future research should establish prospective cohort or randomized controlled trial studies to evaluate treatment outcomes across different patient phenotypes and develop predictive models to optimize patient selection. Additionally, studies incorporating comprehensive morphometric analysis with normative EOM reference data would help clarify the distinct versus overlapping pathological features of MG and TED affecting the EOMs.

## 4 Conclusion

This case report suggests the potential therapeutic benefit of RTX for refractory ocular symptoms in concurrent MG and TED. Quantitative T2 mapping revealed pathologically elevated pre-treatment values that normalized following RTX therapy, providing preliminary objective evidence of treatment response. However, the relative contributions of MG versus TED pathology cannot be definitively separated in this dual autoimmune presentation. These findings suggest that quantitative T2 mapping may serve as a complementary tool for monitoring treatment response in complex overlapping autoimmune orbital disorders, though validation in larger prospective studies is warranted before establishing standardized monitoring protocols.

## Data Availability

The raw data supporting the conclusions of this article will be made available by the authors, without undue reservation.
